# A reduced cell-based phase model for tissue polarity alignment through global anisotropic cues

**DOI:** 10.1038/s41598-017-17611-8

**Published:** 2017-12-12

**Authors:** Kaori Sugimura, Hiroshi Kori

**Affiliations:** Department of Information Sciences, Ochanomizu University, Tokyo, 112-8610 Japan

## Abstract

Ordered polarity alignment of cell populations plays vital roles in biology, such as in hair follicle alignment and asymmetric cell division. Although cell polarity is uniformly oriented along a tissue axis in many tissues, its mechanism is not well understood. In this paper, we propose a theoretical framework to understand the generic dynamical properties of polarity alignment in interacting cellular units, where each cell is described by a reaction–diffusion system, and the cells further interact with one another through the contacting surfaces between them. Using a perturbation method under the assumption of weak coupling between cells, we derive a reduced model in which polarity of each cell is described by only one variable. Essential dynamical properties including the effects of cell shape, coupling heterogeneity, external signal and noise can be clarified analytically. In particular, we show that the anisotropicity of the system, such as oriented cell elongation and axial asymmetry in the coupling strength, can serve as a global cue that drives the uniform orientation of cell polarity along a certain axis. Our study bridges the gap between detailed and phenomenological models, and it is expected to facilitate the study of polarity dynamics in various nonequilibrium systems.

## Introduction

Spatially ordered patterns are ubiquitous in nature and have been of central importance in various disciplines^1–3^. In particular, we are concerned with the dynamical alignment of polarity in nonequilibrium systems of interacting cellular units, including chemical and biological systems, where polarity can be regarded as asymmetric distributions in chemical species within a cellular unit. Polarity has great importance in biology, and it is essential, for example, in cell movement and oriented cell division^4^. A well-known example in biology is planar cell polarity (PCP), which refers to the coordinated alignment of cell polarity across the planar tissue. This results in the formation of the ordered pattern of, e.g., hair follicles and cilia positioning^4^. Although cellular polarity aligns over long distances in skin and wing, its mechanism is not well understood^5^. Recently, several possibilities have been suggested^5–7^. Attributed to the challenge of experimental investigation, theoretical work provides an important role in developing a unifying explanation of the phenomenon. Several mathematical models have been proposed to describe the effects of various factors on polarity alignment, including cell shape, external signal and noise. Some studies employ detailed models, where each cell is described by a reaction–diffusion system, and these cells are further coupled by contacting surfaces^8,9^. Other studies employ simple phenomenological models similar to models for magnetisation or synchronisation^6,10^, which is a reasonable approach because the cell alignment process phenomenologically resembles those observed in a population of spins or oscillatory units^11–15^. Detailed models contain several free parameters and are too complicated to provide a general understanding of the process. Nevertheless, phenomenological models are rather arbitrary and may lack essential dynamical features.

**Figure 1:**
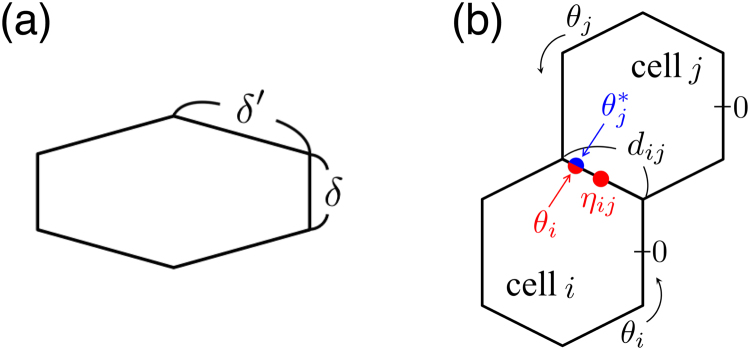
Schematic of (**a**) the cell shape and (**b**) cell alignment. Regular or elongated hexagonal cells are considered with a perimeter of 2*π*, thus 2*δ* + 4*δ*′ = 2*π*. *η*_*ij*_ and *d*_*ij*_ denote the midpoint and the length of the contacting surface between cell *i* and *j*, respectively. ${\theta }_{j}^{\ast }={\theta }_{j}^{\ast }({\theta }_{i})$ denotes a point of *θ*_*j*_ at which cell *j* faces the point *θ*_*i*_ of cell *i*, while *η*_*ij*_ can be regarded as the cell-to-cell direction from cell *i* to cell *j*.

**Figure 2:**
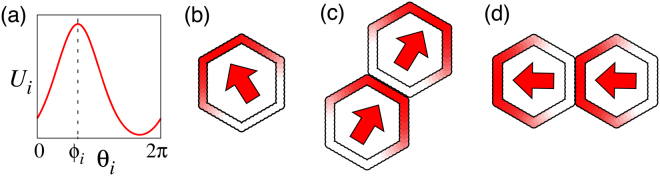
Polarity patterns of (**a**,**b**) single cell and (**c**,**d**) two coupled cells. In (**a**) and (**b**), a steady profile of *U*_*i*_ and its colour scale representation are displayed, respectively. The arrow in (**b**) indicates polarity orientation. In (**c**) and (**d**), typical examples of polarity patterns of two coupled cells with different cell alignments are displayed.

In the present paper, the generic dynamical properties of cell polarity alignment are examined by the derivation and analysis of a reduced model for coupled reaction–diffusion systems. For simplicity, a planar tissue is considered, as in previous studies on PCP^6,8–10^. As a first step, each cell is described by a reaction–diffusion system. The cells mutually inhibit one another through their contacting surfaces, by which polarity alignment occurs between neighbouring cells. It is then shown that a perturbation method under the assumption of weak coupling between cells enables the reduction of the reaction–diffusion model to a phase model, which is drastically simpler than the reaction–diffusion model yet it is a reasonable approximation to it. In particular, the phase model of a simple case, which is a particular case of Eq. (55), can be derived as

1 $${\dot{\varphi }}_{i}=\sum _{j\in A(i)}\{{a}_{ij}\,\sin \,({\varphi }_{j}-{\varphi }_{i})+{c}_{ij}\,\sin \,\mathrm{(2}{\eta }_{ij}-{\varphi }_{i}-{\varphi }_{j})\},$$

where *ϕ*_*i*_ is the phase of cell *i*, which approximately describes the position of the maximum of a reaction–diffusion component, *A*(*i*) is the group of cells adjacent to cell *i*, *η*_*ij*_ is the cell-to-cell direction from cell *i* to cell *j* and *a*_*ij*_ and *c*_*ij*_ are functions of the width *d*_*ij*_ of the contacting surfaces (see Figs 1 and 2). In the absence of the second term, this phase model is same as the model describing spin states in ferromagnets, known as the XY model, and a special case of spatially extended phase oscillator models^16–18^. Such a model was employed in a previous study on PCP^10^. Our phase model is distinct from the study, as it includes novel terms representing geometric information, such as the cell shape and the relative position between neighbouring cells. As the model is easily manageable, essential dynamical properties including the effects of cell shape, external signal and noise can be clarified analytically, which have only been studied numerically in previous works using detailed models^6,8,9^. Finally, we discuss symmetry-breaking patterns in PCP^5–7^ by using of our model. In particular, we point out that axial asymmetry in the system, such as oriented cell elongation and asymmetric distribution of coupling strength, can be a global cue for the orientation of cell polarity across the entire tissue. Our study bridges the gap between detailed and phenomenological models, it is expected to facilitate the study of polarity dynamics in various nonequilibrium systems.

## Reaction–diffusion model

The entire system is composed of a population of planar cells aligned in two-dimensional space. Reaction–diffusion dynamics of each cell takes place on the one-dimensional surface, and the cells further interact with one another through the contacting surfaces between them. For simplicity, it is assumed that every cell has identical shape, which is either regular or elongated hexagonal with a perimeter of 2*π*, and they form hexagonal lattices, as shown in Fig. 1. Each cell obeys the equation,

2 $$\frac{\partial }{\partial t}{{\boldsymbol{X}}}_{i}={\boldsymbol{F}}({{\boldsymbol{X}}}_{i})+\hat{D}\frac{{\partial }^{2}{{\boldsymbol{X}}}_{i}}{\partial {\theta }_{i}^{2}}+\varepsilon \sum _{j\in A(i)}{{\boldsymbol{H}}}_{ij},$$

where ***X***_*i*_ = ***X***_*i*_(*θ*_*i*_, *t*) (*i* = 1, …, *N*) denotes the concentration of chemical species at time *t* and the position *θ*_*i*_ (0 ≤ *θ*_*i*_ < 2*π*) on the surface of each cell, ***F***(***X***_*i*_) describes the local reaction dynamics, $\hat{D}$ is the diagonal matrix consisting of diffusion coefficients, *A*(*i*) is the group of cells adjacent to cell *i*, ***H***_*ij*_ = ***H***_*ij*_(*θ*_*i*_, *t*) describes interactions between cells and *ε* is the coupling strength. Interaction occurs at every contact point and depending on the state at the contact point, i.e.,

3 $${{\boldsymbol{H}}}_{ij}({\theta }_{i},t)={{\boldsymbol{H}}}_{ij}({{\boldsymbol{X}}}_{i}({\theta }_{i},t),{{\boldsymbol{X}}}_{j}({\theta }_{j}^{\ast },t)),$$

where ${\theta }_{j}^{\ast }={\theta }_{j}^{\ast }({\theta }_{i})$ is a point of *θ*_*j*_ at which cell *j* faces the point *θ*_*i*_ of cell *i*, as illustrated in Fig. 1(b), and ***H***_*ij*_(*θ*_*i*_, *t*) vanishes if cell *i* does not contact cell *j* at *θ*_*i*_. As it is described later, external signals and noise may also be considered. In each cell, ***X***_*i*_(*θ*_*i*_, *t*) is assumed to form a unimodal distribution for *ε* = 0; i.e., polarity is spontaneously formed. The orientation of polarity of cell *i* at time *t* is defined by the *θ*_*i*_ value at which the first component of ***X***_*i*_(*θ*_*i*_, *t*), denoted by *U*_*i*_(*θ*_*i*_, *t*), is maximal.

Here, some concrete examples are provided. For ***F*** and $\hat{D}$, two examples are considered: (a) the real Ginzburg–Landau equation (GLE)^19^ and (b) the activator–inhibitor model^20^. Both models have two variables, denoted by ***X***_*i*_ = (*U*_*i*_, *V*_*i*_), and ***F*** and $\hat{D}$ can be written as

4 $${\boldsymbol{F}}=(\begin{array}{c}{U}_{i}-({{U}_{i}}^{2}+{{V}_{i}}^{2}){U}_{i}\\ {V}_{i}-({{U}_{i}}^{2}+{{V}_{i}}^{2}){V}_{i}\end{array}),\quad \hat{D}=(\begin{array}{cc}{D}_{0} & 0\\ 0 & {D}_{0}\end{array}),$$

where *D*_0_ is set to 0.3 at which a stable unimodal distribution is obtained, and

5 $${\boldsymbol{F}}=(\begin{array}{c}\frac{{\rho }_{U}{U}_{i}^{2}}{(1+\kappa {U}_{i}^{2}){V}_{i}}-{\mu }_{U}{U}_{i}+{\sigma }_{U}\\ {\rho }_{V}{U}_{i}^{2}-{\mu }_{V}{V}_{i}\end{array}),\quad \hat{D}=(\begin{array}{cc}{D}_{U} & 0\\ 0 & {D}_{V}\end{array}),$$

where *ρ*_*U*_ = 0.01, *ρ*_*V*_ = 0.02, *μ*_*U*_ = 0.01, *μ*_*V*_ = 0.02, *σ*_*U*_ = 0.0, *κ* = 0.0, *D*_*U*_ = 0.005 and *D*_*V*_ = 0.2, respectively. The parameter values for the latter model are taken from the reference^20^. The former is a long-wave amplitude equation, which is widely used to describe various systems near the onset of instability. The latter is a reaction–diffusion model, describing biological pattern formation^20^. In these models, given appropriate initial conditions, ***X***_*i*_ exhibits a stationary unimodal distribution for *ε* = 0, thus, they are suitable as dynamical models describing cell polarity. Figure 2(a,b) shows a steady profile of *U*_*i*_(*θ*_*i*_,*t*) for *ε* = 0 numerically obtained using the activator–inhibitor model given by equation (5).

As a simple example of intercellular interaction, we consider a linear coupling given by

6 $${{\boldsymbol{H}}}_{ij}({\theta }_{i},t)={S}_{ij}({\theta }_{i})(\begin{array}{c}{U}_{i}({\theta }_{i},t)-{U}_{j}({\theta }_{j}^{\ast },t)\\ 0\end{array}),$$

where *S*_*ij*_ = 1 if cell *i* faces cell *j* at *θ*_*i*_ and *S*_*ij*_ = 0 otherwise; i.e.,

7 $${S}_{ij}({\theta }_{i})=\{\begin{array}{cc}1 & \,{\rm{for}}\,|{\theta }_{i}-{\eta }_{ij}|\, < \,\frac{{d}_{ij}}{2}\,,\,\\ 0 & \,{\rm{otherwise}}{\rm{.}}\,\end{array}$$

The coupling given by equation (6) acts as mutual inhibition between neighbouring cells through the *U*-component for *ε* > 0, causing polarity ordering, as shown in Fig. 2(c,d). Later, another type of linear coupling, i.e., $-{U}_{j}({\theta }_{j}^{\ast },t)$ instead of ${U}_{i}({\theta }_{i},t)-{U}_{j}({\theta }_{j}^{\ast },t)$, is considered to show the robustness of our results.

## Derivation of the phase model

Our reaction–diffusion model given by equation (2) consists of *N* × *M* partial differential equations, where *N* and *M* are the numbers of cells and variables in each cell. For such a model, both analytical and numerical treatments are difficult. Therefore, we applied a perturbation method to equation (2) under the assumption of weak coupling to obtain a phase model, which consists of *N* ordinary differential equations and can be useful for both analytical and numerical analyses. Our method is based on the well-known phase reduction theory^13^, and it is an application of the recently developed method for oscillatory patterns reported in refs.^21,22^.

Let ***X*** ^S^(*θ*) be the stationary distribution of a cell in the unperturbed system (*ε* = 0). Because of the translational symmetry, ***X*** ^S^(*θ* − *θ*_0_) with any constant *θ*_0_ is also a steady solution. The phase *ϕ*_*i*_(*t*) of ***X***_*i*_(*θ*_*i*_, *t*) is defined such that ***X***_*i*_(*θ*_*i*_, *t*) converges to ***X*** ^S^(*θ*_*i*_ − *ϕ*) as *t* → ∞ in the unperturbed system. In other words, ***y***_*i*_(*θ*_*i*_, *t*) → 0 as *t* → ∞ for *ε* = 0, where the deviation ***y***_*i*_(*θ*_*i*_, *t*) is defined by

8 $${{\boldsymbol{X}}}_{i}({\theta }_{i},t)={{\boldsymbol{X}}}^{{\rm{S}}}({\theta }_{i}-{\varphi }_{i})+{{\boldsymbol{y}}}_{i}({\theta }_{i},t),$$

with *ϕ*_*i*_(*t*) being the phase of state ***X***_*i*_(*θ*_*i*_, *t*). Without the loss of generality, we assume that *U* ^S^(*θ*), which is the *U* component of ***X*** ^S^(*θ*), takes its maximum at *θ* = 0. Then, for sufficiently small ***y***_*i*_(*θ*_*i*_, *t*), *ϕ*_*i*_(*t*) of ***X***_*i*_(*θ*_*i*_, *t*) is well approximated by the maximum of *U*_*i*_(*θ*_*i*_, *t*), i.e.,

9 $${\varphi }_{i}(t)\approx {{\rm{argmax}}}_{{\theta }_{i}}{U}_{i}({\theta }_{i},t\mathrm{).}$$

Thus, *ϕ*_*i*_ can be regarded as the orientation of polarity of cell *i*.

The linear operator ${\mathcal L}$ is defined by

10 $$ {\mathcal L} =J+\hat{D}\frac{{\partial }^{2}}{\partial {\theta }^{2}}$$

with Jacobian *J* = ∂***F***(***X***)/∂***X*** determined at ***X*** = ***X*** ^S^(*θ*). The adjoint operator ${ {\mathcal L} }^{\dagger }$ is defined such that it satisfies $\langle {\boldsymbol{A}}, {\mathcal L} {\boldsymbol{B}}\rangle =\langle { {\mathcal L} }^{\dagger }{\boldsymbol{A}},{\boldsymbol{B}}\rangle$, where the inner product of the 2*π*-periodic functions, ***A***(*θ*) and ***B***(*θ*) is defined by

11 $$\langle {\boldsymbol{A}},{\boldsymbol{B}}\rangle ={\int }_{0}^{2\pi }{\boldsymbol{A}}\cdot {\boldsymbol{B}}d\theta \mathrm{.}$$

For equation (2), it can be shown that

12 $${ {\mathcal L} }^{\dagger }={J}^{{\rm{T}}}+\hat{D}\frac{{\partial }^{2}}{\partial {\theta }^{2}},$$

where *J* ^T^ is the transpose of *J*. The eigenfunctions of ${\mathcal L}$ and ${ {\mathcal L} }^{\dagger }$ are denoted by ${{\boldsymbol{Y}}}_{\ell }(\theta )$ and ${{\boldsymbol{Z}}}_{\ell }(\theta )$ $(\ell =\mathrm{0,}\,\mathrm{1,}\,\ldots )$, respectively. In particular, the zero eigenfunctions are denoted by ***Y***_0_ and ***Z***_0_, i.e., ${\mathcal L} {{\boldsymbol{Y}}}_{0}={ {\mathcal L} }^{\dagger }{{\boldsymbol{Z}}}_{0}\mathrm{=0}$. Here, we choose

13 $${{\boldsymbol{Y}}}_{0}=-\frac{\partial {{\boldsymbol{X}}}^{{\rm{S}}}}{\partial \theta }\mathrm{.}$$

These eigenfunctions are assumed to form a complete orthonormal system and are normalised as

14 $$\langle {{\boldsymbol{Z}}}_{\ell },{{\boldsymbol{Y}}}_{m}\rangle ={\delta }_{\ell m}\mathrm{.}$$

The deviation ***y***_*i*_ can be expanded as

15 $${{\boldsymbol{y}}}_{i}({\theta }_{i},t)=\sum _{\ell =1}^{\infty }{{\boldsymbol{C}}}_{\ell }(t){{\boldsymbol{Y}}}_{\ell }({\theta }_{i}-{\varphi }_{i}),$$

where *ϕ*_*i*_ is the phase of state ***X***_*i*_(*θ*_*i*_, *t*). Note that ***Y***_0_(*θ*_*i*_ − *ϕ*_*i*_) is absent in this expansion because ***y***_*i*_(*θ*_*i*_, *t*) → 0 as *t* → ∞ for *ε* = 0.

Substituting equation (8) into equation (2), we obtain

16 $${{\boldsymbol{Y}}}_{0}({\theta }_{i}-{\varphi }_{i}){\dot{\varphi }}_{i}+{\dot{{\boldsymbol{y}}}}_{i}= {\mathcal L} {{\boldsymbol{y}}}_{i}+\varepsilon \sum _{j\in A(i)}{{\boldsymbol{H}}}_{ij}+O({\varepsilon }^{2}\mathrm{).}$$

Taking the inner product with ***Z***_0_(*θ*_*i*_ − *ϕ*_*i*_) and omitting *O*(*ε* ^2^), we finally obtain the phase model as

17 $${\dot{\varphi }}_{i}=\varepsilon \sum _{j\in A(i)}{{\rm{\Gamma }}}_{ij}({\varphi }_{i},{\varphi }_{j}),$$

18 $${{\rm{\Gamma }}}_{ij}=\langle {{\boldsymbol{Z}}}_{0}({\theta }_{i}-{\varphi }_{i}),{{\boldsymbol{H}}}_{ij}^{{\rm{S}}}\rangle ,$$

where ${{\boldsymbol{H}}}_{ij}^{{\rm{S}}}={{\boldsymbol{H}}}_{ij}\{{{\boldsymbol{X}}}^{{\rm{S}}}({\theta }_{i}-{\varphi }_{i}),{{\boldsymbol{X}}}^{{\rm{S}}}({\theta }_{j}^{\ast }-{\varphi }_{j})\}$. Given the functional forms of ***X*** ^S^(*θ*) and ***Z***_0_(*θ*), equation (17) provides a closed equation for the phases *ϕ*_*i*_ (*i* = 1, …, *N*).

It is convenient to express Γ_*ij*_ in terms of Fourier coefficients, ${u}_{k},{z}_{k},{s}_{k}^{(ij)}\in {\mathbb{R}}$, defined by

19 $${U}^{{\rm{S}}}(\theta )=\sum _{k=-\infty }^{\infty }{u}_{k}\,\cos \,k\theta ,$$

20 $${Z}_{0}^{(U)}(\theta )=\sum _{k=-\infty }^{\infty }-{z}_{k}\,\sin \,k\theta ,$$

and

21 $${S}_{ij}(\theta +{\eta }_{ij})=\sum _{k=-\infty }^{\infty }{s}_{k}^{(ij)}\,\cos \,k\theta ,$$

where it is assumed that *S*_*ij*_(*θ* + *η*_*ij*_), *U* ^S^(*θ*) and ${Z}_{0}^{(U)}(\theta )$ are even, even and odd functions, respectively. By substituting these expansions into equation (18) with ***H***_*ij*_ given by equation (6), we obtain a general expression:

22 $${{\rm{\Gamma }}}_{ij}=2\pi \sum _{k,l}{z}_{k}{u}_{l}[{(-\mathrm{1)}}^{l}{s}_{l-k}^{(ij)}\,\sin \,\{(k+l){\eta }_{ij}-k{\varphi }_{i}-l{\varphi }_{j}\}-{s}_{-k-l}^{(ij)}\,\sin \,\{(k+l)({\eta }_{ij}-{\varphi }_{i})\}]\mathrm{.}$$

For the regular and elongated hexagonal cell shapes shown in Fig. 1, we have ${s}_{k}^{(ij)}=\frac{1}{k\pi }\,\sin \,\frac{k{d}_{ij}}{2}(k\ne 0),{s}_{0}^{(ij)}=\frac{{d}_{ij}}{2\pi }$. The coefficients *u*_*k*_ and *z*_*k*_ can be obtained for a given model.

For the GLE, the phase reduction is analytically performed. By solving

23 $${\boldsymbol{F}}({{\boldsymbol{X}}}^{{\rm{S}}})+\hat{D}\frac{{\partial }^{2}}{\partial {\theta }^{2}}{{\boldsymbol{X}}}^{{\rm{S}}}=\mathrm{0,}$$

we obtain

24 $${{\boldsymbol{X}}}^{{\rm{S}}}=({U}^{S},{V}^{S})=\sqrt{1-{D}_{0}}(\cos \,\theta ,\,\sin \,\theta ),$$

thus,

25 $${{\boldsymbol{Y}}}_{0}=(-\frac{d{U}^{S}}{d\theta },-\frac{d{V}^{S}}{d\theta })=\sqrt{1-{D}_{0}}(\sin \,\theta ,-\cos \,\theta )\mathrm{.}$$

Furthermore, by solving ${ {\mathcal L} }^{\dagger }{{\boldsymbol{Z}}}_{0}=0$ with the normalisation 〈***Z***_0_,***Y***_0_〉 = 1, where ${ {\mathcal L} }^{\dagger }= {\mathcal L}$ in the GLE, we obtain

26 $${{\boldsymbol{Z}}}_{0}=({Z}_{0}^{(U)},{Z}_{0}^{(V)})=\frac{1}{2\pi \sqrt{1-{D}_{0}}}(\sin \,\theta ,-\cos \,\theta )$$

**Figure 3:**
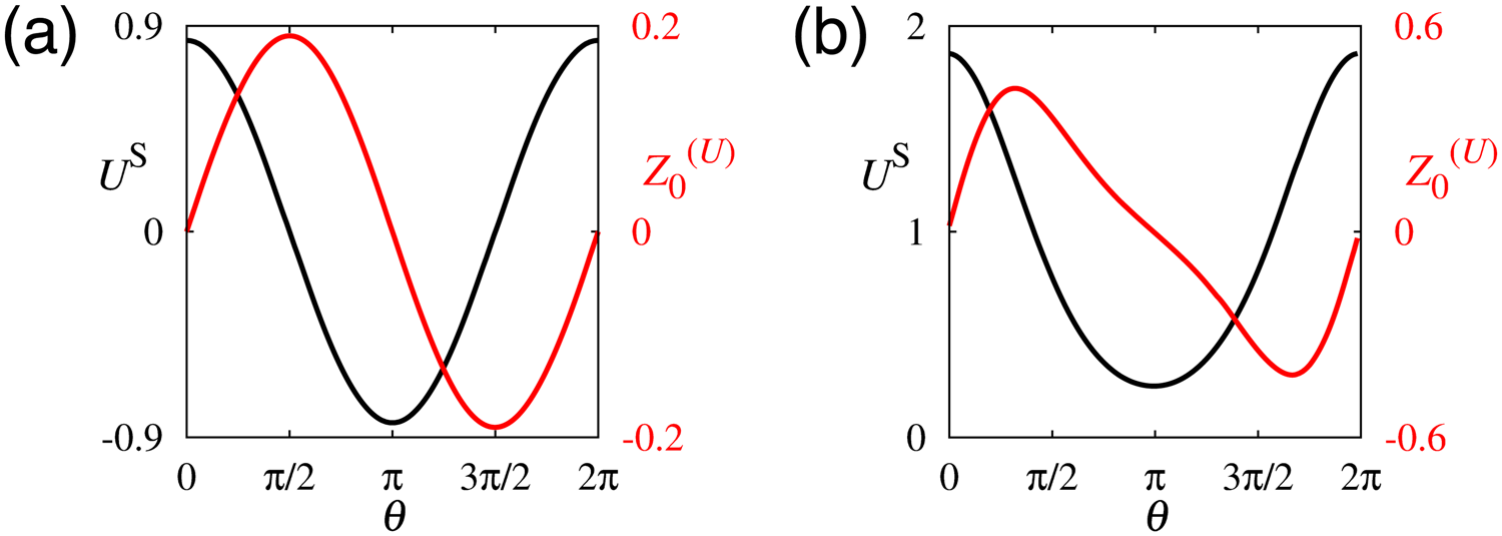
Profile of the steady state *U* ^S^(*θ*) (black lines) and the phase sensitivity function ${Z}_{0}^{(U)}(\theta )$ (red lines) for (**a**) the GLE and (**b**) the activator–inhibitor model.

Functions ***X*** ^S^ and ***Z***_0_ are shown in Fig. 3. Therefore, equation (17) with equation (22) reduces to

27 $${\dot{\varphi }}_{i}=\varepsilon \{{a}_{ij}\,\sin \,({\varphi }_{j}-{\varphi }_{i})+{b}_{ij}\,\sin \,\mathrm{2(}{\eta }_{ij}-{\varphi }_{i})+{c}_{ij}\,\sin \,\mathrm{(2}{\eta }_{ij}-{\varphi }_{i}-{\varphi }_{j})\},$$

where

28 $${a}_{ij}={b}_{ij}=\frac{\sin \,{d}_{ij}}{4\pi },{c}_{ij}=\frac{{d}_{ij}}{4\pi }.$$

In the GLE ***Z***_0_ is proportional to ***Y***_0_ because the linear operator is self-adjoint, i.e. ${ {\mathcal L} }^{\dagger }= {\mathcal L}$, in this particular model. Expressions for all other eigenfunctions are known^23^, although we only need the expressions for ***Z***_0_ and ***Y***_0_ here.

For most models, phase reduction is performed numerically by solving equation (2) for *ε* = 0 and its adjoint equation ${\dot{{\boldsymbol{Z}}}}_{0}={ {\mathcal L} }^{\dagger }{{\boldsymbol{Z}}}_{0}$ with 〈***Z***_0_, ***Y***_0_〉 = 1^22^. For the activator–inhibitor model, *U* ^S^ and ${Z}_{0}^{(U)}$ are obtained, as shown in Fig. 3(b). Their Fourier coefficients are given approximately as *u*_0_ = 0.925, *u*_1_ = 0.397, *u*_2_ = 0.065, *z*_1_ = −0.180, *z*_2_ = −0.062, and the rest of coefficients are negligibly small. We obtain Γ_*ij*_ by substituting these values into equation (22). Then, our phase model becomes

29 $$\begin{array}{rcl}{\dot{\varphi }}_{i} & \simeq  & \varepsilon \{0.124\sin ({\varphi }_{j}-{\varphi }_{i})+0.124\,\sin \,\mathrm{2(}{\eta }_{ij}-{\varphi }_{i})+0.150\,\sin \,\mathrm{(2}{\eta }_{ij}-{\varphi }_{i}-{\varphi }_{j})\\  &  & +0.049\,\sin \,\mathrm{(3}{\eta }_{ij}-2{\varphi }_{i}-{\varphi }_{j})+0.049\,\sin \,({\eta }_{ij}-{\varphi }_{i})\\  &  & +0.033\,\sin \,({\eta }_{ij}-2{\varphi }_{i}+{\varphi }_{j})+0.033\,\sin \,\mathrm{3(}{\eta }_{ij}-{\varphi }_{i})\\  &  & -0.0234\,\sin \,\mathrm{(3}{\eta }_{ij}-{\varphi }_{i}-2{\varphi }_{j})-0.0234\,\sin \,({\eta }_{ij}-{\varphi }_{i})\\  &  & +0.0156\,\sin \,({\eta }_{ij}+{\varphi }_{i}-2{\varphi }_{j})+0.0156\,\sin \,\mathrm{3(}{\eta }_{ij}-{\varphi }_{i})\\  &  & -0.008\,\sin \,\mathrm{(4}{\eta }_{ij}-2{\varphi }_{i}-2{\varphi }_{j})-0.003\,\sin \,\mathrm{2(}{\varphi }_{j}-{\varphi }_{i})-0.003\,\sin \,4({\eta }_{ij}-{\varphi }_{i})\}\mathrm{.}\end{array}$$

**Figure 4:**
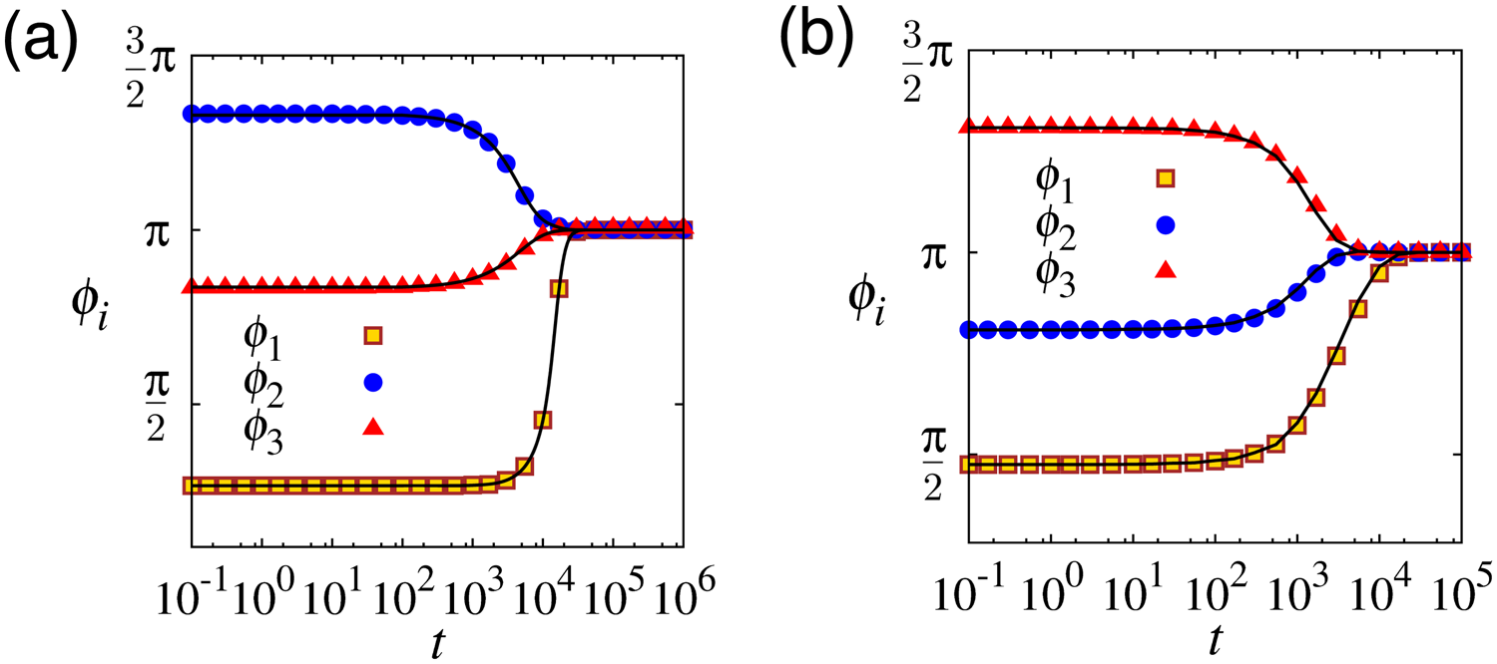
Comparison of the time series obtained from the reaction–diffusion models (symbols) and the corresponding phase models (lines). (**a**) GLE. (**b**) Activator–inhibitor model. In this case, three regular hexagonal cells are aligned in a row, i.e., *η*_12_ = *η*_23_ = 0, *η*_21_ = *η*_32_ = *π*, ${a}_{ij}={b}_{ij}=\frac{\sin \,{d}_{ij}}{4\pi }=\frac{\sqrt{3}}{8\pi },{c}_{ij}=\frac{{d}_{ij}}{4\pi }=\frac{1}{12}$ with *ε* = 0.001.

As shown in Fig. 4, we confirmed the accuracy of our reduction theory for both the GLE and the activator–inhibitor models by comparing the time series of the original model given by equation (2) and that of the phase model given by equation (17) with the corresponding Γ_*ij*_.

It should be noted that the phase sensitivity function ***Z***_0_(*θ*) is very useful in understanding the response of the orientation of polarity to perturbation. See Fig. 3(a) as an example. If the *U* variable is perturbed upwards at *θ* = *π*/2, *ϕ* increases because ${{\boldsymbol{Z}}}_{0}(\pi \mathrm{/2)}\, > \,0$, i.e., the pattern eventually shifts to the right.

## General properties of the phase model

We focus on the phase model given by equation (27) below for the following considerations. If *U* ^S^(*θ*) and ${Z}_{0}^{(U)}(\theta )$ are nearly harmonic, i.e., *u*_*k*_ and *z*_*k*_ with *k* ≥ 2 are small, an approximation for equation (27) can be obtained with

30 $${a}_{ij}={b}_{ij}=-4\pi {z}_{1}{u}_{1}{s}_{2}^{(ij)},{c}_{ij}=-4\pi {z}_{1}{u}_{1}{s}_{0}^{(ij)}.$$

This is actually the case in our activator–inhibitor model: In the coupling function derived from the activator–inhibitor model given by equation (29) the first three terms are considerably larger than the other terms. Thus, the dynamical properties of the activator–inhibitor model are expected to be similar to those of the phase model in equation (27).

We consider different cell alignments and investigate the effect of cell alignments and boundaries on the existence and stability of polarity patterns. Moreover, we investigate the effect of noise and external signals. Finally, we discuss the robustness of our results. Henceforth, without the loss of generality we set *ε* = 1 in numerical simulations.

### Straight cell alignments

We first consider two coupled cells aligned horizontally, as shown in Fig. 2(d). For this alignment, we have *η*_12_ = 0, *η*_21_ = *π*, *a*_12_ = *a*_21_, *b*_12_ = *b*_21_, *c*_12_ = *c*_21_. For convenience, we introduce *ξ* = *ϕ*_1_ + *ϕ*_2_ and *ζ* = *ϕ*_1_ − *ϕ*_2_. From equation (27), we obtain

31 $$\dot{\xi }=-2\varepsilon ({b}_{ij}\,\cos \,\zeta +{c}_{ij})\sin \,\xi ,$$

32 $$\dot{\zeta }=-2\varepsilon ({a}_{ij}+{b}_{ij}\,\cos \,\xi )\sin \,\zeta .$$

Thus, the in-phase state (*ϕ*_1_, *ϕ*_2_) = (*ϕ* ^*^, *ϕ* ^*^) with *ϕ* ^*^ = 0, *π* and *ϕ* ^*^ = $\pm \frac{{\rm{\pi }}}{2}$ are steady. By introducing Δ*ξ* = *ξ* − 2*ϕ* ^*^ and linearizing equations (31) and (32) for small Δ*ξ* and *ζ*, we obtain

33 $$\dot{{\rm{\Delta }}\xi }=-2\varepsilon ({b}_{ij}+{c}_{ij})(\cos \,2{\varphi }^{\ast }){\rm{\Delta }}\xi ,$$

34 $$\dot{\zeta }=-2\varepsilon ({a}_{ij}+{b}_{ij}\,\cos \,2{\varphi }^{\ast })\zeta \mathrm{.}$$

The solutions (*ϕ*_1_, *ϕ*_2_) = (0, 0) and (*π*, *π*) are thus linearly stable when

35 $$\varepsilon ({a}_{ij}+{b}_{ij}) > 0\,\,{\rm{a}}{\rm{n}}{\rm{d}}\,\,\varepsilon ({b}_{ij}+{c}_{ij}) > 0.$$

In this case, the solution $({\varphi }_{1},{\varphi }_{2})=\pm (\frac{\pi }{2},\frac{\pi }{2})$ is unstable. The GLE with *ε* > 0 satisfies this condition. For the 1D straight chain of any number *N* of cells with open and periodic boundaries, we obtain the same stability condition for the in-phase state *ϕ*_*i*_ = 0 and *ϕ*_*i*_ = *π* for 1 ≤ *i* ≤ *N*, which can be shown by applying the Gershgorin circle theorem to the corresponding stability matrix.

### Effects of cell alignments, cell shapes and heterogeneity in coupling strengths

We investigated the dependence of polarity pattern on complex cell alignments and cell shapes, as well as the heterogeneity in coupling strengths. It should be pointed out that in equation (27), the second and third terms facilitate the phase *ϕ*_*i*_ and the mean phase $\frac{{\varphi }_{i}+{\varphi }_{j}}{2}$ to be oriented to the cell-to-cell direction *η*_*ij*_, respectively. If only the first term is present in equation (27), which is the case in the XY model, there is a family of stable solutions *ϕ*_*i*_ = *ϕ* ^*^ (*i* = 1, …, *N*) with arbitrary *ϕ* ^*^ values, and the realised polarity pattern is determined by the initial conditions. However, if either *b*_*ij*_ or *c*_*ij*_ is nonvanishing, the in-phase state even with a particular *ϕ* ^*^ value does not exist except for special networks such as a straight chain.

**Figure 5:**
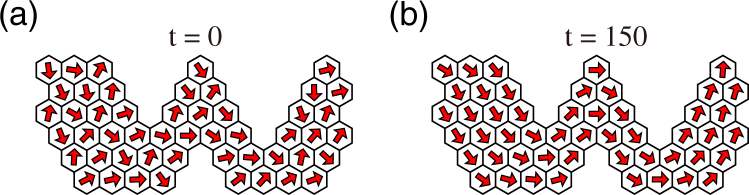
Polarity pattern for winding cell alignment with a regular hexagonal shape, obtained numerically by the phase model given by equation (27). (**a**) Initial and (**b**) final patterns. Each arrow indicates the phase of each cell. Initial conditions were chosen such that no topological defects appeared (see Methods).

To obtain useful insight into the dynamical behaviour of a complicated alignment of cells, we made an approximation in the phase model using the assumption that the neighbouring cells are nearly in phase. Under the approximation that *ϕ*_*i*_ = *ϕ*_*j*_ for any neighbouring cells (i.e., the in-phase state), equation (27) reduces to

36 $${\dot{\varphi }}_{i}=\varepsilon {R}_{i}\,\sin \,\mathrm{2(}\overline{{\eta }_{i}}-{\varphi }_{i}),$$

where *R*_*i*_ > 0 and $\overline{{\eta }_{i}}\in {\mathbb{R}}$ are determined by

37 $${R}_{i}{e}^{{\rm{i}}2\overline{{\eta }_{i}}}=\sum _{j\in A(i)}({b}_{ij}+{c}_{ij}){e}^{i2{\eta }_{ij}},$$

which can be interpreted as the effective strength and the preferred direction of the net interaction of cell *i*, respectively. For hexagonal lattices with each cell shape being regular hexagonal, *R*_*i*_ vanishes for cell *i* that does not facing boundaries of the lattice because *b*_*ij*_ and *c*_*ij*_ are not depending on *i*, *j* and *η*_*ij*_ takes the values 0, 2*π*/*n*, 4*π*/*n*, …, 2(*n* − 1)*π*/*n* with *n* = 6. The same is true for square lattices with each cell shape being square. Nevertheless, for cells at the boundary, *R*_*i*_ is non-vanishing and $\overline{{\eta }_{i}}$ it is approximately parallel to the boundary line. Therefore, cell polarity at the boundary tends to be parallel to the boundary line and cell polarity of bulk cells is smoothly aligned to that of the neighbouring cells. As shown in Fig. 5, this prediction is confirmed using the system with winding cell alignment.

When the cell shape is elongated, *R*_*i*_ is non-vanishing even in the bulk. In this case, $\overline{{\eta }_{i}}$ tends to orient to the direction of a contact surface with a larger width. When the number of bulk units is much more than that of boundary units, polarity orientation is dominantly depending on the cell shape. In particular, when the cell shape is uniformly elongated as shown in Fig. 1(a), stability analysis is straightforward. In this case, equation (36) reduced to

38 $${\dot{\varphi }}_{i}=-2\varepsilon \lambda \,\sin \,2{\varphi }_{i},$$

where

39 $$\lambda =\frac{\sin \,\delta }{4\pi }+\frac{\delta }{4\pi }-2(\frac{\sin (\frac{\pi -\delta }{2})}{4\pi }+\frac{\pi -\delta }{8\pi })\sin \,\frac{\delta }{2}.$$

**Figure 6:**
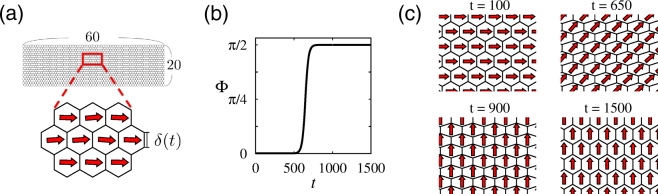
Polarity pattern for planar alignment in a periodic system of 60 × 20 cells, obtained numerically by the phase model given by equation (27). In (**a**), initial cell shape (regular hexagonal, $\delta (0)=\frac{\pi }{3}$) and initial phases are shown. In (**b**), the time series of the mean phase Φ(*t*) defined as $Q(t){e}^{i{\rm{\Phi }}(t)}=\frac{1}{N}{\sum }_{j}{e}^{i{\varphi }_{j}(t)}$ with *Q* ≥ 0 and ${\rm{\Phi }}\in {\mathbb{R}}$ is shown. Cells are elongated only for 500 ≤ *t* < 1000 during which $\delta (t)=\frac{\pi }{3}-\frac{\pi }{10}$; otherwise $\delta (t)=\frac{\pi }{3}$. In (**c**) snapshots are presented. Initial conditions were chosen such that no topological defects appeared (see Methods).

Thus, stability depends on the sign of *λ*. For *λ* > 0 (*λ* < 0), which is the case for $d > \frac{\pi }{3}$ $(d < \frac{\pi }{3})$, states *ϕ*_*i*_ = 0 or *ϕ*_*i*_ = *π* $({\varphi }_{i}=\frac{\pi }{2}\,{\rm{or}}\,{\varphi }_{i}=-\frac{\pi }{2})$ for all *i* are stable. For *λ* = 0, which is the case for $d=\frac{\pi }{3}$, *ϕ*_*i*_ dynamics becomes neutral, and the steady state is determined by the initial conditions. To clearly demonstrate the effect of cell elongation, a two-dimensional periodic system was considered, as shown in Fig. 6. Initially, the cells were set to be regular hexagonal. Because the boundary effects are negligibly small for the periodic boundary condition under consideration, phases can be aligned with arbitrary values determined by the initial conditions. Here, a random initial condition was used, where phases were chosen from a uniform distribution within the range (−0.5, 0.5). At *t* = 100, phases were almost perfectly aligned at *ϕ*_*i*_ ≈ 0, which is approximately the average of the initial phases. At *t* = 500, the cell shape was changed to be elongated. Then, the cell polarity was aligned upwards, pointing to the direction of a contact surface with a larger width, as predicted above. This polarity pattern was maintained even when the cell shape was returned to be regular hexagonal (*t* > 1000).

A similar result can be obtained by considering heterogeneity in coupling strength even for regular hexagonal cell shapes. We considered the condition in which coupling strength *ε* in our reaction–diffusion model given by equation (2) is dependent on *i*, *j*, by replacing *ε* with *ε*(1 + *α*_*ij*_). Then, the corresponding phase model becomes

40 $${\dot{\varphi }}_{i}=\varepsilon \sum _{j\in A(i)}(1+{\alpha }_{ij})\{a\,\sin ({\varphi }_{j}-{\varphi }_{i})+b\,\sin \,2({\eta }_{ij}-{\varphi }_{i})+c\,\sin (2{\eta }_{ij}-{\varphi }_{i}-{\varphi }_{j})\},$$

where $a=b=\frac{\sqrt{3}}{8\pi }$ and $c=\frac{1}{12}$. By assuming an in-phase state, this equation reduces to

41 $${\dot{\varphi }}_{i}=\varepsilon (b+c)\sum _{j\in A(i)}(1+{\alpha }_{ij})\sin \,2({\eta }_{ij}-{\varphi }_{i}).$$

**Figure 7:**
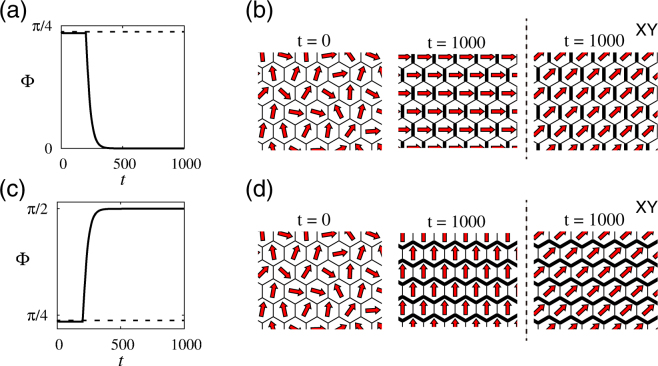
Polarity pattern in the system with heterogeneous coupling strengths. The cell alignment is the same as that of Fig. 6, while cell shape is fixed to be regular hexagonal. The coupling strength is initially homogeneous, i.e., *α*_*ij*_ = 0 for 0 ≤ *t* < 200. For *t* ≥ 200, we set *α*_*ij*_ = 0.1 and −0.1 for the surfaces with *η*_*ij*_ = 0, *π* in (**a**), (**b**) and (**c**), (**d**), respectively; and *α*_*ij*_ = 0 otherwise. Thus, the contacting surfaces shown with bold lines have larger coupling strength. (**a**,**c**) Time series of the mean phase Φ(*t*) obtained with the phase model given by equations (27) (solid lines) and with its XY model variant (dashed lines) in which *b*_*ij*_ and *c*_*ij*_ values are set to zero, while ${a}_{ij}=\frac{\sqrt{3}}{8\pi }$ is unchanged. (**b**,**d**) Snapshots are shown. The rightmost panels show snapshots obtained by the XY model variant. The same initial condition was used for all cases.

Now we introduce an axial asymmetry such that only the surfaces along the vertical axis, which are shown as bold lines in Fig. 7, have *α*_*ij*_ = *α*, and *α*_*ij*_ = 0 for other surfaces. In this case, we further obtain

42 $${\dot{\varphi }}_{i}=-2\varepsilon \alpha (b+c)\sin \,2{\varphi }_{i}.$$

Thus, the sign of *α* plays the exactly same role as that of *λ* in equation (38); the polarity pattern is aligned along the axis with stronger coupling. In Fig. 7, numerical results can be seen obtained using our phase model with coupling heterogeneity, given by equation (40). To emphasise the effects of geometry-dependent terms, we also show results obtained using equation (40) with *b* and *c* being set to zero, corresponding to the XY model, which indeed shows no response to the coupling heterogeneity. The axial asymmetry of the system under consideration affects the dynamics only in the presence of geometric-dependent terms.

### Effects of noise and external signals

Phase reduction is also possible when our reaction–diffusion model includes external signals and noise, given as

43 $$\frac{\partial }{\partial t}{{\boldsymbol{X}}}_{i}={\boldsymbol{F}}({{\boldsymbol{X}}}_{i})+\hat{D}\frac{{\partial }^{2}}{\partial {\theta }_{i}^{2}}{{\boldsymbol{X}}}_{i}+\varepsilon \sum _{j\in A(i)}{{\boldsymbol{H}}}_{ij}+{\varepsilon }_{{\rm{e}}}{{\boldsymbol{G}}}_{i}+{{\boldsymbol{p}}}_{i},$$

where ***G***_*i*_ = ***G***_*i*_(*θ*_*i*_, *t*) is the external signal, *ε*_e_ is its strength, and ${{\boldsymbol{p}}}_{i}=({p}_{i}^{(1)}({\theta }_{i},t),{p}_{i}^{(2)}({\theta }_{i},t),\ldots )$ is white Gaussian noise that satisfies ${\rm{E}}[{p}_{i}^{(m)}(\theta ,t)]=0$ and ${\rm{E}}[{p}_{i}^{(m)}(\theta ,t){p}_{j}^{(n)}(\theta ^{\prime} ,t^{\prime} )]={\nu }_{m}{\delta }_{ij}{\delta }_{mn}\delta (\theta -\theta ^{\prime} )\delta (t-t^{\prime} )$ with E[·] representing the expected value, and *ν*_*m*_ is the noise intensity. For sufficiently small *ε*_e_ and *ν*_*m*_, the same procedure was followed as for equation (2) to obtain

44 $${\dot{\varphi }}_{i}=\varepsilon \sum _{j\in A(i)}{{\rm{\Gamma }}}_{ij}({\varphi }_{i},{\varphi }_{j})+{\varepsilon }_{e}{{\rm{\Pi }}}_{i}({\varphi }_{i},t)+{q}_{i}(t)$$

where

45 $${{\rm{\Pi }}}_{i}({\varphi }_{i})=\langle {{\boldsymbol{Z}}}_{0}({\theta }_{i}-{\varphi }_{i}),{{\boldsymbol{G}}}_{i}({\theta }_{i},t)\rangle ,$$

46 $${q}_{i}(t)=\langle {{\boldsymbol{Z}}}_{0}({\theta }_{i}-{\varphi }_{i}),{{\boldsymbol{p}}}_{i}({\theta }_{i},t)\rangle \mathrm{.}$$

Here, *q*_*i*_(*t*) is Gaussian white noise that satisfies

47 $${\rm{E}}[{q}_{i}(t)]=0,\quad {\rm{E}}[{q}_{i}(t){q}_{j}(t^{\prime} )]=\nu {\delta }_{ij}\delta (t-t^{\prime} ),$$

where $\nu ={\sum }_{m}{\nu }_{m}{\int }_{0}^{2\pi }d\theta {\{{Z}^{(m)}(\theta )\}}^{2}$ (see Methods).

In the case of GLE, any generic choice of external signal ***G***_*i*_(*θ*_*i*_, *t*) yields

48 $${{\rm{\Pi }}}_{i}={c}_{i}(t)\sin ({\psi }_{i}(t)-{\varphi }_{i})$$

because ***Z***_0_(*θ*) contains only the first harmonics. As a simple example, we consider a unimodal distribution peaked at *θ*_*i*_ = *ψ*(*t*), given as

49 $${{\boldsymbol{G}}}_{i}({\theta }_{i})=(\cos (\psi (t)-{\theta }_{i}),0),$$

and we obtain

50 $${{\rm{\Pi }}}_{i}=\frac{1}{2\sqrt{1-{D}_{0}}}\,\sin (\psi -{\varphi }_{i}).$$

The phase model under consideration is actually a gradient system, i.e.,

51 $${\dot{\varphi }}_{i}=-\frac{\partial }{\partial {\varphi }_{i}} {\mathcal H} +{q}_{i}$$

with the potential function ${\mathcal H} = {\mathcal H} (\{{\varphi }_{i}\})$ given by

52 $$\begin{array}{rcl} {\mathcal H}  & = & -\frac{\varepsilon }{2}\sum _{i}\sum _{j\in A(i)}\{{a}_{ij}\,\cos ({\varphi }_{j}-{\varphi }_{i})+{b}_{ij}\,\cos \,2({\eta }_{ij}-{\varphi }_{i})+{c}_{ij}\,\cos (2{\eta }_{ij}-{\varphi }_{i}-{\varphi }_{j})\}\\  &  & -{\varepsilon }_{e}\sum _{i}\frac{1}{2\sqrt{1-{D}_{0}}}\,\cos (\psi -{\varphi }_{i}).\end{array}$$

**Figure 8:**
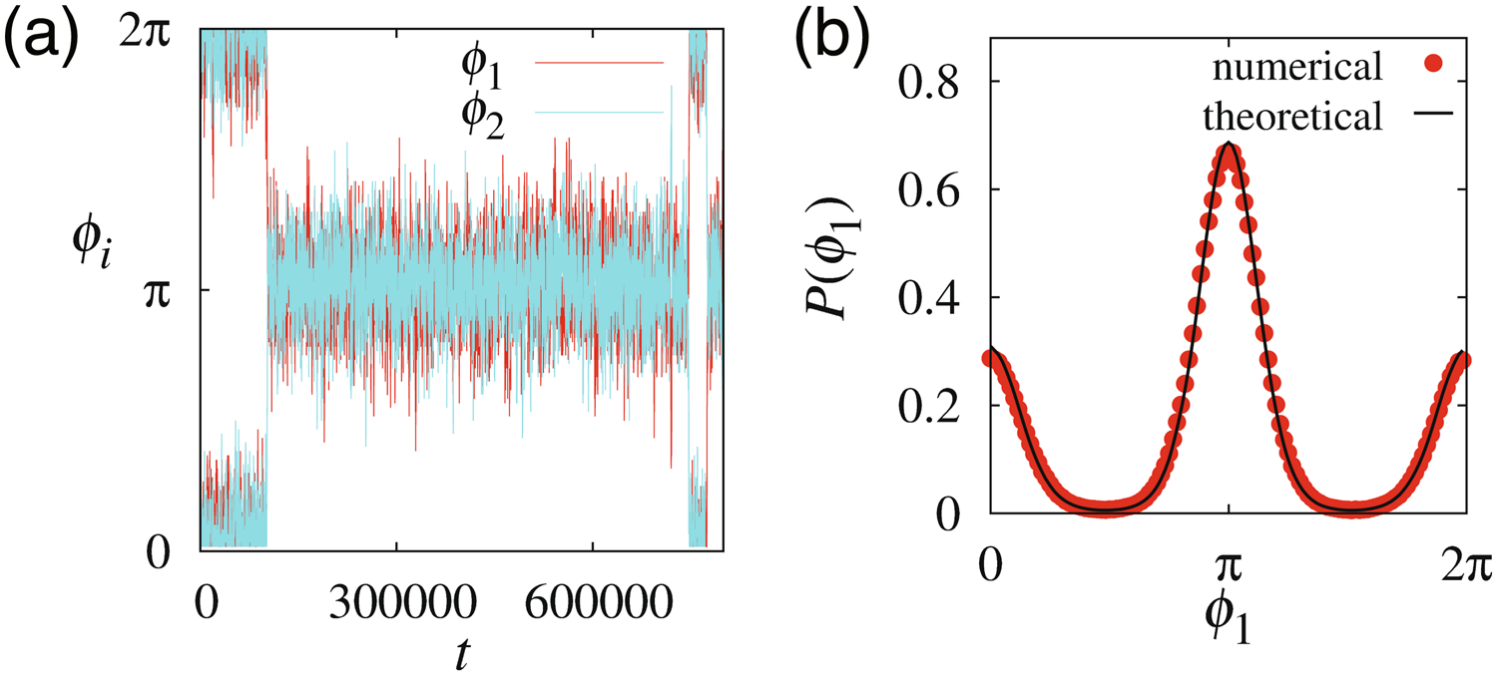
Polarity orientation in two coupled cells in the presence of external signals and noise. (**a**) Time series. (**b**) The probability density function obtained numerically and theoretically.

Thus, we obtained a probability distribution

53 $$P(\{{\varphi }_{i}\})=C\exp \,[-\frac{2 {\mathcal H} (\{{\varphi }_{i}\})}{\nu }],$$

where *C* is the normalisation constant. As shown in Fig. 8, the probability distribution obtained numerically from the GLE, given by equations (43) and (4), is in good agreement with equation (53).

### Robustness

We discuss the robustness of our results described above against changes in our model equations. Our numerical simulations for regular hexagonal cell shapes were performed using equation (27) with ${a}_{ij}={b}_{ij}=\frac{\sin \,{d}_{ij}}{4\pi }$ and ${c}_{ij}=\frac{{d}_{ij}}{4\pi }$ where ${d}_{ij}=\frac{\pi }{3}$. We verified that these results do not change qualitatively for small changes in *a*_*ij*_, *b*_*ij*_, *c*_*ij*_ values. Qualitative change is certainly expected when the stability condition given by equation (35) is disturbed.

We also observed that there is no qualitative difference between the phase models reduced from the GLE and the activator–inhibitor model, as shown in Fig. 4. This suggests that higher harmonics in Γ_*ij*_ does not considerably affect dynamics, at least when they are small.

We can also consider different types of couplings in our reaction–diffusion model other than that in equation (6). For example, we considered

54 $${{\boldsymbol{H}}}_{ij}({\theta }_{i},t)={S}_{ij}({\theta }_{i})(\begin{array}{c}-{U}_{j}({\theta }_{j}^{\ast },t)\\ 0\end{array}),$$

which describes mutual inhibition, as well as equation (6). By assuming *U* ^S^(*θ*) and ${Z}_{0}^{(U)}(\theta )$ are nearly harmonic, we obtain the following approximation

55 $${\dot{\varphi }}_{i}=\varepsilon \{{a}_{ij}\,\sin ({\varphi }_{j}-{\varphi }_{i})+{b^{\prime} }_{ij}\,\sin ({\eta }_{ij}-{\varphi }_{i})+{c}_{ij}\,\sin (2{\eta }_{ij}-{\varphi }_{i}-{\varphi }_{j})\},$$

where ${a}_{ij}=-4\pi {z}_{1}{u}_{1}{s}_{2}^{(ij)},{b^{\prime} }_{ij}=4\pi {z}_{1}{u}_{0}{s}_{1}^{(ij)},{c}_{ij}=-4\pi {z}_{1}{u}_{1}{s}_{0}^{(ij)}$. The phase model given by (55) is again a gradient system with the potential function given by

56 $$ {\mathcal H} =-\frac{\varepsilon }{2}\sum _{i}\sum _{j\in A(i)}\{{a}_{ij}\,\cos ({\varphi }_{j}-{\varphi }_{i})+2{b^{\prime} }_{ij}\,\cos ({\eta }_{ij}-{\varphi }_{i})+{c}_{ij}\,\cos (2{\eta }_{ij}-{\varphi }_{i}-{\varphi }_{j})\}.$$

The second term in equation (55) differs from that in equation (27), thus different dynamical properties may appear. However, in case of GLE given by equation (4), we obtain ${a}_{ij}=\frac{\sin \,{d}_{ij}}{4\pi },{b^{\prime} }_{ij}=0,{c}_{ij}=\frac{{d}_{ij}}{4\pi }$. Thus, the corresponding phase model is a special case of (27) where *b*_*ij*_ = 0. It is straightforward to confirm that the existence and stability analysis performed above does not change in this case.

## Discussion and Conclusion

A theoretical framework for understanding the general dynamical properties of the alignment process of cellular polarity has been proposed. We derived the phase model as a reduced model of coupled reaction–diffusion systems and investigated polarity dynamics using the phase model. The first term of our phase model facilitates the polarity ordering between interacting cells, which is the same as the in-phase coupling in the Kuramoto model and the ferromagnetic coupling in the XY model^18^. The remaining terms include geometric information of the system, i.e., cell-to-cell directions between neighbouring cells and the width of contacting surfaces. Therefore, our phase model exhibits polarity dynamics that depends on the shape of individual cells and the alignment of cell populations, as well as heterogeneity in coupling strengths. In particular, we show that the axial asymmetry of the system facilitates the formation of globally oriented polarity patterns.

The advantages of our method are substantial. Whereas our reaction–diffusion model given in equation (2) is an *N*- set of reaction–diffusion systems with multiple variables, this complicated system is reduced to an *N*-dimensional system of ordinary differential equations as given in equation (17). Using the steady state of the unperturbed reaction–diffusion system, we obtain the coupling function Γ_*ij*_, by which we can perform various analytical and numerical analyses, which are presented in this paper. Although the studied phenomena are nonlinear, our framework enables us to obtain analytical results even in the presence of noise.

Finally, we discuss the symmetry-breaking patterns of cell polarity in biological tissues^5–7^ with the help of our model. As reviewed by Aw and Devenport^5^, although PCP aligns over long distances in skin and wing, the global cues that orient tissue polarity are not well understood. This review highlights two plausible choices. One is a factor expressed in tissue-wide gradients along the axis of polarity, supported by experimental and theoretical studies [see list of references in^5^]. The other is mechanical tension applied to the tissues, which may act over long distances. Aw *et al*. recently reported that in mouse skin, an axial asymmetry in a PCP component (Celsr1, an atypical cadherin) emerges during the process of mechanical deformation along the anterior–posterior (AP) axis; i.e., the concentration of Celsr1 is higher on the junctions perpendicular to the AP axis. They demonstrated that such Celsr1 asymmetry emerges spontaneously during neighbour exchange, because the junctions perpendicular to the AP axis are persistent and there is sufficient time for Celsr1 to accumulate on those junctions, whereas other junctions are nascent. They speculated that such axial asymmetry contributes to the formation of polar asymmetry, as indeed developed in the skin. As also reviewed by Aw and Devenport^5^, in the Drosophila wing, a similar axial asymmetry in PCP components is formed during the process of mechanical deformation, and cell polarity is eventually aligned along the AP axis^6^. Our model can provide an understanding of how the axial asymmetry in the system contributes to the formation of globally aligned patterns of cell polarity. Because PCP components are essential to cell-to-cell communication for polarity alignment, the concentrations of PCP components can naturally be associated with the coupling strength; i.e., coupling is expected to be stronger on cell-to-cell junctions with higher concentration of PCP components. Therefore, we interpret the vertical axis of Fig. 7(b) as the AP axis, with which polarity is eventually aligned. This symmetry-breaking phenomenon does not emerge in the XY model, i.e., equation (27) with *b*_*ij*_ = 0 and *c*_*ij*_ = 0, because in that case, the model has rotational symmetry even when asymmetry in the coupling strength is considered. The symmetry-breaking phenomenon occurs in our phase model because it has geometry-dependent interaction terms originating from geometry-dependent interactions in our reaction–diffusion model. Further investigation on the robustness of global alignment against randomness in cell shapes and coupling strengths is required. For this aim, we need to extend our theoretical framework to arbitrary cell shapes, and investigations along this line are in progress.

## Methods

### Numerical implementation and visualisation

In Fig. 2, we obtained *U*_*i*_(*θ*, *t*) in steady state by numerically solving the equations of the reaction–diffusion model given by equations (2) and (5). The arrows indicate *θ*_*i*_ at which *U*_*i*_(*θ*_*i*_, *t*) is maximal.

Figure 3(a) shows the analytical results, given by equations (24) and (26), whereas Fig. 3(b) shows the numerical results obtained using the activator–inhibitor model, given by equations (2) and (5), for *ε* = 0 and its adjoint equation ${\dot{{\boldsymbol{Z}}}}_{0}={ {\mathcal L} }^{\dagger }{{\boldsymbol{Z}}}_{0}$ with 〈***Z***_0_,***Y***_0_〉 = 1.

Figure 4 shows the numerical results: Solid lines were obtained from the reaction–diffusion models, given by equations (2), (4), and (5). Symbols were obtained from the phase models, given by equations (27) and (29). For the reaction–diffusion models, the phase *ϕ*_*i*_(*t*) was numerically determined as follows. Firstly, the first Fourier component of *U*_*i*_(*θ*,*t*) was calculated as

57 $${\hat{U}}_{i}(t)=\frac{1}{2\pi }{\int }_{0}^{2\pi }{U}_{i}(\theta ,t){e}^{-i\theta }d\theta .$$

Then, the phase *ϕ*_*i*_(*t*) was given as the solution to

58 $${\hat{U}}_{i}(t)=C(t){e}^{-i{\varphi }_{i}(t)},$$

where *C*(*t*) ≥ 0 and *ϕ*_*i*_(*t*) are real. In this way, *θ*_*i*_ = *ϕ*_*i*_(*t*) approximately coincides with the maximum of *U*_*i*_(*θ*_*i*_) when *U*_*i*_(*θ*_*i*_) is nearly harmonic because

59 $${U}_{i}({\theta }_{i},t)\approx C(t)\cos ({\theta }_{i}-{\varphi }_{i}(t)).$$

Figures 5–7 show the numerical results obtained by the phase model. Initial phases were taken randomly from a uniform distribution $(-\frac{\pi }{2},\frac{\pi }{2}),(-0.5,0.5)$ and $(\frac{\pi }{4}-\frac{\pi }{2},\frac{\pi }{4}+\frac{\pi }{2})$ in Figs 5–7, respectively. For such initial conditions, no defect in the polarity patterns appears.

In Fig. 8, numerical results were obtained from the reaction–diffusion model given by equations (43) and (4) with an inclusion of additive noise ${{\boldsymbol{p}}}_{i}({\theta }_{i},t)=({p}_{i}^{\mathrm{(1)}}\mathrm{,0)}$ and external signal ***G***_*i*_ = (cos(*ψ* − *θ*_*i*_), 0) with *ψ* = *π*. The phase was determined in the same manner as Fig. 4. The theoretical probability distribution was obtained using $P({\varphi }_{1})={\int }_{0}^{2\pi }P({\varphi }_{1},{\varphi }_{2})d{\varphi }_{2}$, where *P*(*ϕ*_1_, *ϕ*_2_) is given by equation (53). The two cells were aligned vertically, as shown in Fig. 2(d), thus *η*_12_ = 0 and *η*_21_ = *π*. Other parameter values were *ν*_1_ = *ν*_2_ = 0.005, *ε*_e_ = 0.0002 and *D*_0_ = 0.2.

### Calculation of equation (47)

Equation (47) is obtained as follows.

60 $$\begin{array}{rcl}{\rm{E}}[{q}_{i}(t)] & = & {\rm{E}}\,[{\int }_{0}^{2\pi }d\theta Z(\theta -{\varphi }_{i})\cdot {{\boldsymbol{p}}}_{i}(t)d\theta ]\end{array}$$

61 $$\begin{array}{rcl} & = & {\rm{E}}\,[{\int }_{0}^{2\pi }d\theta \sum _{m}{Z}^{(m)}(\theta -{\varphi }_{i}){p}_{i}^{(m)}d\theta ]\end{array}$$

62 $$\begin{array}{rcl} & = & {\int }_{0}^{2\pi }d\theta \sum _{m}{Z}^{(m)}(\theta -{\varphi }_{i}){\rm{E}}[{p}_{i}^{(m)}]d\theta \end{array}$$

63 $$\begin{array}{rcl} & = & 0,\end{array}$$

and

64 $$\begin{array}{rcl}{\rm{E}}[{q}_{i}(t){q}_{j}(t^{\prime} )] & = & {\rm{E}}[\int {\int }_{0}^{2\pi }d\theta d\theta ^{\prime} \{{\boldsymbol{Z}}(\theta -{\varphi }_{i}(t))\cdot {{\boldsymbol{p}}}_{i}(t)\}\{{\boldsymbol{Z}}(\theta ^{\prime} -{\varphi }_{j}(t^{\prime} ))\cdot {{\boldsymbol{p}}}_{j}(t^{\prime} )\}]\end{array}$$

65 $$\begin{array}{rcl} & = & {\rm{E}}\,[\int {\int }_{0}^{2\pi }{\int }_{0}^{2\pi }d\theta d\theta ^{\prime} \{\sum _{m}{Z}^{(m)}(\theta -{\varphi }_{i}(t)){p}_{i}^{(m)}(t)\}\{\sum _{m^{\prime} }{Z}^{(m^{\prime} )}(\theta ^{\prime} -{\varphi }_{j}(t^{\prime} )){p}_{j}^{(m^{\prime} )}(t^{\prime} )\}]\end{array}$$

66 $$\begin{array}{rcl} & = & {\rm{E}}\,[\int {\int }_{0}^{2\pi }d\theta d\theta ^{\prime} \sum _{m,m^{\prime} }{Z}^{(m)}(\theta -{\varphi }_{i}(t)){p}_{i}^{(m)}{Z}^{(m^{\prime} )}(\theta ^{\prime} -{\varphi }_{j}(t^{\prime} )){p}_{j}^{(m^{\prime} )}(t^{\prime} )]\end{array}$$

67 $$\begin{array}{rcl} & = & \int {\int }_{0}^{2\pi }d\theta d\theta ^{\prime} \sum _{m,m^{\prime} }{Z}^{(m)}(\theta -{\varphi }_{i}(t)){Z}^{(m^{\prime} )}(\theta ^{\prime} -{\varphi }_{j}(t^{\prime} )){\rm{E}}[{p}_{i}^{(m)}(t){p}_{j}^{(m^{\prime} )}(t^{\prime} )]\end{array}$$

68 $$\begin{array}{rcl} & = & \int {\int }_{0}^{2\pi }d\theta d\theta ^{\prime} \sum _{m,m^{\prime} }{Z}^{(m)}(\theta -{\varphi }_{i}(t)){Z}^{(m^{\prime} )}(\theta ^{\prime} -{\varphi }_{j}(t^{\prime} )){\nu }_{m}{\delta }_{ij}{\delta }_{mm^{\prime} }\delta (\theta -\theta ^{\prime} )\delta (t-t^{\prime} )\end{array}$$

69 $$\begin{array}{rcl} & = & {\int }_{0}^{2\pi }d\theta \sum _{m}{\nu }_{m}{\{{Z}^{(m)}(\theta -{\varphi }_{i}(t))\}}^{2}\end{array}$$

70 $$\begin{array}{rcl} & = & \sum _{m}{\nu }_{m}{\int }_{0}^{2\pi }d\theta {\{{Z}^{(m)}(\theta )\}}^{2}\mathrm{.}\end{array}$$

## References

[1] Cross M, Hohenberg P (1993). Pattern formation outside of equilibrium. Rev. Mod. Phys..

[2] Meinhardt H, Gierer A (2000). Pattern formation by local self-activation and lateral inhibition. Bioessays.

[3] Nakao H, Mikhailov AS (2010). Turing patterns in network-organized activator-inhibitor systems. Nature Physics.

[4] Devenport D (2014). The cell biology of planar cell polarity. The Journal of cell biology.

[5] Aw WY, Devenport D (2017). Planar cell polarity: global inputs establishing cellular asymmetry. Current opinion in cell biology.

[6] Aigouy B (2010). Cell flow reorients the axis of planar polarity in the wing epithelium of drosophila. Cell.

[7] Aw WY, Heck BW, Joyce B, Devenport D (2016). Transient tissue-scale deformation coordinates alignment of planar cell polarity junctions in the mammalian skin. Current Biology.

[8] Amonlirdviman K (2005). Mathematical modeling of planar cell polarity to understand domineering nonautonomy. Science.

[9] Burak Y, Shraiman BI (2009). Order and stochastic dynamics in drosophila planar cell polarity. PLoS Comput Biol.

[10] Ayukawa T (2014). Dachsous-dependent asymmetric localization of spiny-legs determines planar cell polarity orientation in drosophila. Cell reports.

[11] Winfree AT (1967). Biological rhythms and the behavior of populations of coupled oscillators. Journal of Theoretical Biology.

[12] Kosterlitz J (1974). The critical properties of the two-dimensional xy model. Journal of Physics C: Solid State Physics.

[13] Kuramoto, Y. *Chemical Oscillations, Waves, and Turbulence* (Springer, New York 1984).

[14] Winfree, A. T. *The Geometry of Biological Time* (Springer, New York 2001), 2nd edn.

[15] Pikovsky, A., Rosenblum, M. & Kurths, J. *Synchronization: A Universal Concept in Nonlinear Sciences* (Cambridge Univ. Press 2001).

[16] Sakaguchi H, Shinomoto S, Kuramoto Y (1988). Mutual Entrainment in Oscillator Lattices with Nonvariational Type Interaction. Prog. Theo. Phys..

[17] Ermentrout G (1992). Stable periodic solutions to discrete and continuum arrays of weakly coupled nonlinear oscillators. SIAM Journal on Applied Mathematics.

[18] Acebrón JA, Bonilla LL, Vicente CJP, Ritort F, Spigler R (2005). The Kuramoto model: A simple paradigm for synchronization phenomena. Reviews of modern physics.

[19] Aranson IS, Kramer L (2002). The world of the complex Ginzburg-Landau equation. Rev. Mod. Phys..

[20] Koch A, Meinhardt H (1994). Biological pattern formation: from basic mechanisms to complex structures. Reviews of Modern Physics.

[21] Nakao H, Yanagita T, Kawamura Y (2014). Phase-reduction approach to synchronization of spatiotemporal rhythms in reaction-diffusion systems. Physical Review X.

[22] Kawamura Y, Nakao H (2015). Phase description of oscillatory convection with a spatially translational mode. Physica D: Nonlinear Phenomena.

[23] Tuckerman LS, Barkley D (1990). Bifurcation analysis of the eckhaus instability. Physica D: Nonlinear Phenomena.

